# The Role of Peroxisome Proliferator-Activated Receptors and Their Transcriptional Coactivators Gene Variations in Human Trainability: A Systematic Review

**DOI:** 10.3390/ijms19051472

**Published:** 2018-05-15

**Authors:** Miroslav Petr, Petr Stastny, Adam Zajac, James J. Tufano, Agnieszka Maciejewska-Skrendo

**Affiliations:** 1 Faculty of Physical Education and Sport, Charles University, 162 52 Prague, Czech Republic; petr@ftvs.cuni.cz (M.P.); tufano@ftvs.cuni.cz (J.J.T.); 2 Department of Theory and Practice of Sport, The Jerzy Kukuczka Academy of Physical Education in Katowice, 40-065 Katowice, Poland; a.zajac@awf.katowice.pl; 3 Faulty of Physical Education, Gdansk University of Physical Education and Sport, 80-336 Gdansk, Poland; maciejewska.us@wp.pl

**Keywords:** human performance, aerobic training, genetic predisposition, anaerobic threshold, muscle fibers, glucose tolerance, insulin response, VO_2_max, VO_2_peak, mitochondria activity, cholesterol levels

## Abstract

Background: The peroxisome proliferator-activated receptors (*PPARA*, *PPARG*, *PPARD*) and their transcriptional coactivators’ (*PPARGC1A*, *PPARGC1B*) gene polymorphisms have been associated with muscle morphology, oxygen uptake, power output and endurance performance. The purpose of this review is to determine whether the PPARs and/or their coactivators’ polymorphisms can predict the training response to specific training stimuli. Methods: In accordance with the Preferred Reporting Items for Systematic Reviews and Meta Analyses, a literature review has been run for a combination of PPARs and physical activity key words. Results: All ten of the included studies were performed using aerobic training in general, sedentary or elderly populations from 21 to 75 years of age. The non-responders for aerobic training (VO_2_peak increase, slow muscle fiber increase and low-density lipoprotein decrease) are the carriers of *PPARGC1A* rs8192678 Ser/Ser. The negative responders for aerobic training (decrease in VO_2_peak) are carriers of the *PPARD* rs2267668 G allele. The negative responders for aerobic training (decreased glucose tolerance and insulin response) are subjects with the *PPARG* rs1801282 Pro/Pro genotype. The best responders to aerobic training are *PPARGC1A* rs8192678 Gly/Gly, *PPARD* rs1053049 TT, *PPARD* rs2267668 AA and *PPARG* rs1801282 Ala carriers. Conclusions: The human response for aerobic training is significantly influenced by PPARs’ gene polymorphism and their coactivators, where aerobic training can negatively influence glucose metabolism and VO_2_peak in some genetically-predisposed individuals.

## 1. Introduction

Although sport scientists strive to conceive of experiments that investigate the effects of specific diets or training strategies, their findings may not agree across general populations and specific groups of athletes. Even when an experiment is meticulously designed using homogeneous samples, the outcomes of a study can largely differ between individuals within a homogeneous group. As these inter-individual responses are often masked when reporting the mean values of dependent variables, some researchers have suggested that sport science should refrain from reporting mean data and should focus on individual data. Although this solution may seem logical, internal independent factors should also be considered when analyzing and discussing inter-individual differences in response to interventions.

As each individual might respond differently to the same external stimuli, the effectiveness of an intervention can likely be somewhat explained by an athlete’s genetic make-up. For example, some individuals may exhibit minimal changes in a specific dependent variable, but others who undergo the exact same intervention may experience a massive improvement for the same variable. Using the famous HERITAGE study as an example, researchers demonstrated significant individual responses in VO_2_max following 20 weeks of aerobic training [1] and, under same conditions, even negative metabolic responses manifested with common blood markers (systolic blood pressure, insulin, triacylglycerol, HDL cholesterol) in 8.4–13.3% of negative responders [2].

Peroxisome proliferator-activated receptor (PPAR) proteins belong to the steroid hormone receptor superfamily and combine with the retinoid X receptors to form heterodimers that regulate genes involved in lipid and glucose metabolism, adipocyte differentiation, fatty acid transport, carcinogenesis and inflammation [3,4]. PPARs exist in three different forms as PPAR-alpha (PPARα), PPAR-beta/delta (PPARβ/δ) and PPAR-gamma (PPARγ), which are encoded by the genes *PPARA*, *PPARD* and *PPARG*. PPARα and PPARβ/δ are present mainly in the liver and in tissues with high levels of fatty acid oxidation such as skeletal muscle, cardiac muscle and the kidneys. PPARγ are predominantly active in adipocytes affecting their differentiation and growth, and they are also an interesting target for pharmacotherapy of diabetes mellitus type 2 (DM2).

Peroxisome proliferator-activated receptor gamma, coactivator 1 alpha (PGC1α), encoded by the *PPARGC1A* gene, is a transcriptional coactivator of the PPAR superfamily. This protein interacts with PPARγ, which enables its interaction with many others transcriptional factors. PGC1α is involved in mitochondrial biogenesis, glucose utilization, fatty acid oxidation, thermogenesis, gluconeogenesis and insulin signaling [5]. Peroxisome proliferator-activated receptor gamma, coactivator 1 beta (PGC1β), encoded by the *PPARGC1B* gene, together with the *PPARGC1A* gene, encodes homologous proteins that, through nuclear transcription factor coactivation, regulate adipogenesis, insulin signaling, lipolysis, mitochondrial biogenesis, angiogenesis and hepatic gluconeogenesis [5].

Human performance is a multifactorial domain where genetic predisposition may act as a key intrinsic factor. Of the many genes that have been studied in relation to sport performance and exercise, *PPARA*, *PPARG*, *PPARD* and their transcriptional coactivators’ *PPARGC1A* and *PPARGC1B* gene polymorphisms have been associated with elite athletic performance, which has been related to muscle morphology [6], oxygen uptake [7,8], power output [9] and endurance performance [6]. Therefore, identifying links between PPARs (and their coactivators) and human performance may shed light on the possibility of identifying athletes with specific genetic sporting potential, possibly also leading to genetically-specialized training methods for athletes who carry specific PPAR gene variants.

From various sets of single nucleotide polymorphisms (SNPs), the PPAR signaling pathway has been reported as one the most related to human cellular bioenergetics and VO_2_max trainability in the genome-wide association study (GWAS) [10]. Previous reviews found that PPARs and/or their coactivators’ genes were associated with endurance performance [11,12,13] or improvements in weight reduction following training programs [14]; however, their relation to training responses has not been determined yet.

Since there many studies showing the association between PPARs and/or their coactivators’ gene polymorphisms and human performance, it is beneficial to also know the relationship between PPARs and different training responses. Therefore, the purpose of this review is to summarize whether the PPARs and/or their coactivators’ polymorphisms can predict human response to specific training stimuli. We hypothesized that PPARs and/or their coactivators’ gene polymorphisms can predict the response to aerobic and anaerobic training, that the PPARs and/or their coactivators’ polymorphisms can predict the amount of appropriate training load and eventually can determine the responders to specific training methods (e.g., hypoxia-training).

## 2. Results

**Figure 1:**
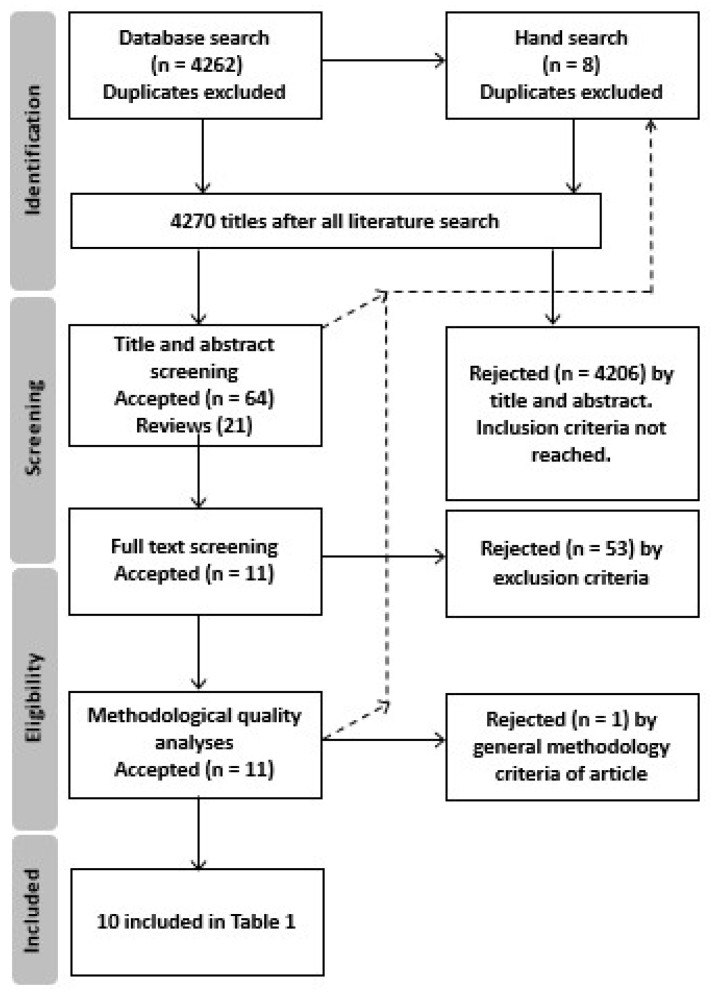
Review flow chart for articles included in tables.

The literature search resulted in a total of 7389 articles, which was reduced to 4262 after removing duplicates. The number of eligible articles was further reduced to 64 after screening article titles and abstracts to include PPARs and/or their coactivators’ gene polymorphisms in relation to physical activity (Figure 1). Of these studies, 53 were rejected following full-text screening, and one was rejected based on the methodological quality criteria. Finally, 10 studies (Table 1) were included in the analysis.

None of the 10 included studies were performed on elite athletes, using resistance training, using maximum training load or any other specific training method. One study [15] focusing on elite athlete response for resistance training and regarding the amount of training load had to be rejected due to a lack of a methodological approach, specifically a lack of reproducibility. Because this study showed a significant role of *ACE*, *ACTN3* and *PPARGC1A* genes with the volumes of specific training loads within the training process macrostructure of elite weightlifters [15], we suggest that PPARs’ role in resistance training and elite sport should be studied in further research. On the other hand, all of the included studies were performed using aerobic training in general, sedentary or elderly populations from 21 to 75 years of age (Table 1), and one study did not find any association with *PPARGC1A* and trainability [16]. Therefore, our hypotheses have been confirmed only in the case of training responses to aerobic training in non-athletic populations. One study using a GWAS design [10] has been rejected by Exclusion Criterion 4.

The response to aerobic training is partly described in *PPARGC1A*, *PPARG* and *PPARD* in various aerobic training approaches referring to the improvement of training performance and the response of glucose metabolism and insulin sensitivity (Table 2).

In the range of the population included in the study, we can state that PPARs and/or their coactivators’ gene polymorphisms may be able to predict the human response to aerobic training at moderate intensities up to the lactate threshold. Specifically, the PPARs and their coactivators’ gene polymorphisms can predict high response, no response or even negative response for aerobic training estimated by glucose tolerance, insulin response, body fat, VO_2_peak, anaerobic threshold, mitochondria activity, cholesterol levels and slow muscle fibers’ increase (Table 2).

The *PPARGC1A* rs8192678 Gly/Gly genotype has been associated with greater increases of an individual’s anaerobic threshold [17], a greater increase of slow muscle fibers [18], greater mitochondria activity [18], a greater decrease of low-density and total lipoprotein cholesterol [19] and a greater VO_2_peak increase after aerobic training than *PPARGC1A* rs8192678 Ser allele carriers. Moreover, *PPARGC1A* rs8192678 Ser allele carriers had no response in slow muscle fibers’ changes, changes in low-density and total lipoprotein cholesterol and VO_2_peak [20] after aerobic training.

*PPARD* rs1053049 TT homozygotes have been associated with greater increases in insulin sensitivity and greater decreases of fasting insulin levels than C allele carriers [17]. *PPARD* rs2267668 AA homozygotes have been found to have greater increases in insulin sensitivity, greater increases of the individual anaerobic threshold and greater increases in VO_2_peak after aerobic training than G allele carriers [17]. Moreover, the *PPARD* rs2267668 G allele carriers have been found to have a negative response (decrease) of VO_2_peak after aerobic training intervention [17]. The *PPARD* rs2016520 T allele carriers have been found to have a greater increase of VO_2_max and maximum power output after aerobic training than CC homozygotes (only in black subjects), and the CC genotype had a higher increase of plasma HDL cholesterol (in white subjects) [21]. The *PPARD* rs2076167 GC genotype had a higher increase of plasma HDL cholesterol (only in white subjects).

*PPARG* rs1801282 Pro/Pro homozygotes have been found to have more decreased fasting insulin [22] than Ala/Pro heterozygotes and more decreased body fat than Ala allele carriers [23] after aerobic training. The Ala carriers have been found to have more increased glucose tolerance [24], more decreased fasting immunoreactive insulin and a more decreased insulin resistance index [25] after aerobic training than Pro/Pro homozygotes. *PPARG* Pro/Pro homozygotes have been found to have a negative response fasting immunoreactive insulin and a more decreased insulin resistance index after aerobic training.

## 3. Discussion

The main finding of this review is that PPARs and their coactivators’ gene polymorphisms may predict the human response to aerobic training at moderate intensities up to the lactate threshold, which might be expected. On the other hand, the lack of research in human training response to anaerobic training and specific training methods indicate that further research I needed. In this manner, there are significant cues that PPARs and their coactivators’ gene polymorphisms can determine the anaerobic training effectiveness in response to training loads [15] (our finding includes also intensity at the anaerobic threshold). Although we had to exclude one study [15] for a lack of reproducibility, we have to highlight the importance of their findings (determination of resistance training load) as a significant suggestion for future research focus. A previous study on compound dinucleotide repeat polymorphism in *ALAS2* intron 7 in Han Chinese males determined that individuals with dinucleotide repeats ≤166 bp compared to individuals with dinucleotide repeats >166 bp were significantly better responders for high altitude training (measured as ∆VO_2_max), especially to living-high exercise-high training-low (HiHiLo) training [26]. This specificity can be considered as the key information for creating a long-term endurance training program. Equally, women of multi-ethnicity origin from the FAMuSS cohort, homozygous for the mutant allele 577X in the *ACNT3* gene, demonstrated greater absolute and relative 1 repetition maximum gains of elbow flexors compared with the homozygous wild type (577RR) after resistance training when adjusted for body mass and age [27]. This review has to note that PPARs are not sufficiently analyzed for such specific training methods, although their connection to aerobic performance has been well known since the HERITAGE study results in 2001 [1].

This review summarized the best responders for aerobic training in relation to PPARs and their coactivators’ genes polymorphisms (*PPARGC1A* rs8192678 Gly/Gly, *PPARD* rs1053049 TT, *PPARD* rs2267668 AA, *PPARD* rs2016520 T allele carriers and *PPARG* rs1801282 Ala allele carriers) in a common population [17,18,20,21]. On the other hand, the evaluation summary on the effects in *PPARD* rs2267668 G allele carriers and *PPARG* rs1801282 Pro/Pro homozygotes showed several negative responses to aerobic training. Most likely, this could be the most important information from exercise genomics studies, i.e., knowledge of genetic markers that can be beneficial for predicting the individual response to training in athletes and normal individuals, that is setting up the parameters of training protocols. On the other hand, the evaluation summary on effects in *PPARD* rs2267668 G allele carriers and *PPARG* rs1801282 Pro/Pro homozygotes showed several negative responses to aerobic training. Although the development of reliable tools for predicting exercise response based on one’s genetic make-up is challenging and undoubtedly requires further research, the mentioned genetic variants seem to identify individuals who are not instructed to use classical aerobic training methods to improve their health or physical performance. Similarly, the non-responders for the *PPARGC1A* rs8192678 polymorphism who were Ser/Ser homozygotes might perform the aerobic training to improve metabolism functions such as mitochondria activity, but without a complex impact on improved health or endurance performance.

As was indicated earlier, post-training increase in aerobic fitness was found to be associated with the presence of a specific *PPARGC1A* rs8192678 Gly allele during a lifestyle intervention [17]. These observations led to the suggestion that the rs8192678 Gly allele may be a key element associated with the efficiency of aerobic metabolism; however, the question of how the rs8192678 Gly and Ser variants affect cardiorespiratory capacity remains. A general explanation is the engagement of the PGC-1α co-activator in the regulation of energy metabolism, as well as mitochondrial biogenesis and function, causing an upregulation of oxidative metabolism and parallel changes in muscle fiber types [28]. More detailed in vitro studies with the use of recombinant plasmids bearing Gly or Ser at position 482 in the PGC-1α protein showed that the PGC-1α 482Ser variant was less efficient as a co-activator of the MEF2C (myocyte enhancer factor 2C), which is a transcription factor especially important in the regulation of glucose transportation in skeletal muscle [29]. MEF2C, when coactivated by the PGC-1α, is particularly involved in the activation of GLUT4 (glucose transporter 4) via direct interaction with this gene promoter, which results in the facilitation of glucose uptake by the cell [30]. The Gly482Ser polymorphic site is located in the domain critical for the binding interaction between MEF2C and PGC-1α proteins, and in this way, the rs8192678 Gly and Ser variants may influence the co-activation process, which may have consequences not only for glucose uptake, glycogen synthesis and the subsequent synthesis of fatty acids, but also for the transformation of muscle fiber type [28]. On the latter point, the expression of genes specific for type I slow-twitch fibers, such as MB (myoglobin) and TNNI1 (troponin I, slow skeletal muscle), is triggered by the calcineurin signaling pathway depending on PGC-1α/MEF2 coactivation [31].

The described structure of the *PPARD* gene differs from the classical eukaryotic gene model: it has been reported to encompass nine exons, of which exons 1–3, the 5′-end of exon 4 and the 3′-end of exon 9 are untranslated [32]. The rs2016520 polymorphic point is located precisely in the 5′UTR region of exon 4 of the *PPARD* gene. In this region, the recognition sites for Sp1 binding were found, raising the suggestion that rs2016520 may interfere with interaction between the *PPARD* gene and the Sp1 transcription factor, affecting in this way the *PPARD* expression level. Such an assumption was confirmed in the in vitro studies showing a higher transcriptional activity for the minor C allele compared with the major T allele of rs2016520 [33], which as a consequence may lead to impairment of PPARδ function and its ability to regulate the energy metabolism in skeletal muscles, in this manner influencing physical performance [34]. As was indicated in our metanalysis, during an intervention exercise training program performed in healthy (but previously sedentary) individuals of the HERITAGE Family Study, rs2016520 CC homozygotes were characterized by a smaller training-induced increase in maximal oxygen consumption and a lower training response in maximal power output compared with the CT and the TT genotypes, both in black and white subjects. Furthermore, CC homozygotes showed the greatest increases in HDL-C (white subjects) and Apo A-1 (black subjects) levels [21]. It was speculated that the greater promoter activity of *PPARD* rs2016520 CC homozygotes could result in higher PPARβ/δ levels. On the other hand, endurance training induces the elevated PPARβ/δ-specific agonists’ availability, and the same ligands also increase the expression of the *ABCA1* gene, which is a key regulator of reverse cholesterol transport. All above-mentioned facts lead to the suggestion that the greatest increases in HDL-C levels observed in *PPARD* rs2016520 CC individuals might result from an increase in *ABCA1* gene expression [21].

Maintaining normal blood glucose levels is considered critical for preventing metabolic syndrome [35], and chronically impaired blood glucose responses comprise a significant risk factor for type II diabetes mellitus (DM2) [36]. Exercise in general has positive effects on glucose metabolism and DM2 prevention [37], thus encouraging individuals who are non-/poor responders to exercise is highly valuable. Our review shows that *PPARG* rs1801282 Ala allele carriers, *PPARD* rs2267668 AA homozygotes and *PPARD* rs1053049 TT homozygotes have, for some reason, more effectively improved glucose sensitivity and related parameters compared to their counterparts (Table 2). As regards the *PPARG* rs1801282 Ala allele, similar findings related to glycemic response to exercise have been found in diabetic patients [38] or in Japanese healthy men [25] who completed three months of supervised aerobic training. The *PPARG* Ala allele showed decreased binding affinity to the cognate promoter element and reduced ability to transactivate responsive promoters [39] and seems to be more responsive not only to exercise, but also to nutritional intervention; a significant decrease of waist circumference in diabetic patients was found following the swap from a normal to a Mediterranean diet [40]. The functional relevance of the Pro12Ala amino acid change in the PPARγ protein results from its localization within the molecule encoded by the *PPARG* gene. Pro12Ala substitution is a consequence of rs1801282 SNP located within the exon B sequence of the *PPARG* gene. This amino acid change is located within the AF-1 domain that controls the ligand-independent activation function of PPARγ. The presence of Ala at position 12 of the PPARγ protein may indirectly facilitate the chemical modification of some amino acid residues (phosphorylation and/or SUMOylation) responsible for decreasing the PPARγ activity as a transcriptional regulator involved in energy control and lipid/glucose homeostasis [41]. Different transcriptional activities of factors bearing Pro or Ala at position 12 in the PPARγ protein were confirmed in in vitro experiments, which recognized the Ala form as less active, characterized by a decreased ability to activate the transcription of appropriate constructs containing PPRE [42] or specific genes [39]. Moreover, analyses performed in vivo also revealed that expression of PPARγ target genes depends on the Pro12Ala genotypes [43]. Genetic association studies, as well as whole-body insulin sensitivity measurements documented that Ala allele carriers displayed a significantly improved insulin sensitivity [44], which may have the consequence of better glucose utilization in working skeletal muscles [45]. The studies investigating the effects of *PPARD* gene variants on glucose homeostasis are only marginal; only the effect of the contribution to the risk of DM2 of nine common variants in *PPARD* (including rs1053049 and rs2267668) in Chinese Hans was found in the rs6902123 polymorphism [46]. Another study also showed that *PPARD* polymorphisms (rs1053049, rs6902123 and rs2267668) could be involved in the development of insulin resistance and DM2 [47].

The combined effect of *PPARD*, *PPARG* and *PPARGC1A* gene polymorphisms on endurance exercise response and on health-related parameters is unclear, due to the amount of analyzed genes. Although, the results of studies included in our review seems to be promising in this manner, an evaluation demands larger cohorts with long-term supervised exercise programs to reach significance. At this moment, any life-style interventional program including exercise in normal people or a training regimen in athletes is not recommended according to the genomic data. On the other hand, the PPARs’ relation to training methods’ responses such as hypoxia [48] and resistance training [15] seems to have high potential to future research.

## 4. Materials and Methods

### 4.1. Review Process

The review was performed according to the Preferred Reporting Items for Systematic Reviews and Meta Analyses (PRISMA) [49] guidelines using the review protocol assigned in International Prospective Register of Systematic Reviews (PROSPERO) under Database No. CRD42018082236. The final articles’ eligibility was assessed using the adapted “Standard Protocol Item Recommendation for Interventional Trials” (SPIRIT) checklist (Supplementary Material 1).

### 4.2. Literature Search

To find articles related to PPAR polymorphisms’ role in physical activity, a systematic computerized literature search was conducted on 20 November 2017, in PubMed (1940 to the search date), Scopus (1823 to the search date) and Web of Science (1974 to the search date). A combination of the following search terms was used: (PPAR) OR (peroxisome AND proliferator AND activated AND receptor) AND (sport) OR (physical AND activity) OR (endurance) OR (exercise) OR (performance) OR (movement). The search did not include comments, proceedings, editorial letters, conference abstracts and dissertations. Reviews were included for a manual search of their reference lists. A manual search of the reference lists of included articles was also performed (Figure 1).

### 4.3. Literature Selection

After identifying potential articles, the titles and abstracts were reviewed by two independent reviewers (Petr Stastny, Miroslav Petr) to select relevant articles for full-text screening. The title and abstract screening focused on four related inclusion criteria:

- Sampling of genetic polymorphisms in the *PPARA*, *PPARG*, *PPARD*, *PPARGC1A* and *PPARGC1B*, genes.
- Analyses of genetic polymorphisms on sport phenotype (markers of sport phenotype) or related physical activity domains (e.g., body mass, fat mass, energy uptake, performance, physical fitness).
- Population of athletes and other healthy populations with a physical activity record.
- Cross-sectional, cohort, case control, intervention, control trials or GWAS.

When the inclusion of articles was questionable, the reviewers came to agreement after a personal discussion. The full texts of relevant articles were then analyzed to determine which were to be used in the final analysis. This full-text screening was performed by three independent reviewers (Petr Stastny, Miroslav Petr, Agnieszka Maciejewska-Skrendo), who also completed the data extraction form (Supplementary Material 2). Data collection was performed in interventional studies only. During the full-text screening, the following exclusion criteria were used:

(1) the full text was not available in English;
(2) the study did not contain an appropriate description of measuring devices, physical activity or genetic sampling procedures;
(3) the study did not include a specification of physical activity;
(4) the study did not report a quantitative performance outcome;
(5) the study did not perform the intervention of a physical training program;
(6) the study was not reproducible by the methodological quality criteria.

## 5. Conclusions

PPARs and their coactivators’ polymorphism genes can predict high response, no response or even negative response for aerobic training estimated by glucose tolerance, insulin response, body fat, VO_2_max, anaerobic threshold, VO_2_peak, mitochondria activity, cholesterol and slow muscle fibers’ increase. Future studies should determine the role of PPARs and their coactivators in anaerobic training and more specific training methods (such as hypoxia) than moderate to lactate threshold aerobic training. The non-responders for aerobic training in VO_2_peak, slow muscle fiber increase and low-density lipoprotein decrease are the carriers of *PPARGC1A* rs8192678 Ser/Ser. The negative responders for aerobic training in VO_2_peak are carriers of the *PPARD* rs2267668 G allele. The negative responders for aerobic training in glucose tolerance and insulin response are carriers of the *PPARG* rs1801282 Pro/Pro genotype. The best responders to aerobic training are *PPARGC1A* rs8192678 Gly/Gly, rs1053049 TT, *PPARD* rs2267668 AA and *PPARG* rs1801282 Ala carriers.

## Figures and Tables

**Table 1:** Basic description of included interventional studies. PPAR = peroxisome proliferator-activated receptor.

Study	Gene/Polymorphism	Population	Aim	Main Result
Stefan et al., 2007 [17]	*PPARGC1A* Gly482Ser (rs8192678) *PPARD* (rs2267668) (rs6902123) (rs2076167) (rs1053049)	German; *n* = 136 (men 63, women 73), Tuebingen Lifestyle Intervention Program. Age 45 ± 1 years, body mass 86.5 ± 1.5 kg	To investigate, whether selected SNPs predict the response of aerobic exercise training on changes in aerobic physical fitness and insulin sensitivity and whether they affect mitochondrial function in human myotubes in vitro.	Genetic variations in *PPARD* and *PPARGC1A* modulate mitochondrial function and changes in aerobic physical fitness and insulin sensitivity during lifestyle intervention.
Steinbacher et al., 2015 [18]	*PPARGC1A* Gly482Ser (rs8192678)	Austrian; *n* = 28 (men only), Salzburg Atherosclerosis Prevention Programme in Subjects at High Individual Risk. Age 59 ± 7 years (range 50–69), body mass 88 ± 2.2 kg	To investigate the myocellular responses in the vastus lateralis muscle of untrained male carriers of this SNP and of a control group after 10 weeks of endurance training.	The single nucleotide polymorphism Gly482Ser in the *PPARGC1A* gene impairs exercise-induced slow-twitch muscle fiber transformation in humans.
Tobina et al., 2017 [19]	*PPARGC1A* Gly482Ser (rs8192678)	Japanese; *n* = 119 (men 49, women 70), all participants >65 years of age. Age 71 ± 6 years, body mass 57.5 ± 9.8 kg	This study investigated the effects of *PPARGC1A* Gly482Ser polymorphisms on alterations in glucose and lipid metabolism induced by 12 weeks of exercise training.	The *PPARGC1A* Gly482Ser polymorphism is associated with the response of low-density lipoprotein cholesterol concentrations following exercise training in elderly Japanese.
Ring-Dimitriou, et al., 2014 [20]	*PPARGC1A* Gly482Ser (rs8192678)	Austrian; *n* = 24 (men only), untrained individuals selected from SAPHIR program. Age 58.3 ± 5.7 years, body mass 87.2 ± 7.6 kg	To test if untrained men who are homozygous or heterozygous carriers of the rare allele in *PPARGC1A* show a reduced change in oxygen uptake and work rate at the submaximal performance level compared to men characterized by the common genotype after 10 weeks of endurance exercise.	Investigated SNP affects the trainability of aerobic capacity measured as VO_2_ or work rate at the respiratory compensation point of previously untrained middle-aged men. The highest responders were Gly/Gly genotypes compared to Gly/Ser and Ser/Ser genotypes.
He et al., 2008 [16]	*PPARGC1A* Thr394Thr (rs17847357) Gly482Ser (rs8192678) A2962G (rs6821591)	Chinese of Han origin; *n* = 102 (men only), soldiers from a local police army. Age 19 ± 1 years, height 171.7 ± 5.8 cm, body mass 60.3 ± 6.5 kg	To examine the possible association between *PPARGC1A* genotypes and both maximal (i.e., VO_2_max) and submaximal endurance capacity (i.e., running economy in a pre-training state (baseline) and after endurance training.	None of the VO_2_max and RE-related traits were associated with the Gly482Ser and Thr394Thr polymorphisms at baseline nor after training. The A2962G polymorphism was however associated with VO_2_max at baseline, as carriers of the G allele (AG1GG genotypes; *n* = 49) had higher levels of VO_2_max than the AA group (*n* = 53).
Weiss et al., 2005 [22]	*PPARG* Pro12Ala (rs1801282)	Caucasian; *n* = 73, (men 32, women 41), healthy sedentary subjects aged 50–75 years.	To investigate whether a common functional gene variant predicts insulin action and whether improvements in insulin action in response to endurance exercise training are associated with *PPARG* Pro12Ala.	Endurance training-induced changes in the insulin response to oral glucose are associated with the *PPARG* Pro12Ala genotype in men, but not in women.
Zarebska et al., 2014 [23]	*PPARG* Pro12Ala (rs1801282)	Polish; *n* = 201 (women only), no history of any metabolic or cardiovascular diseases. Age 21 ± 1 years	To examine the genotype distribution of the *PPARG* Pro12Ala allele in a group of Polish women measured for selected body mass and body composition variables before and after the completion of a 12-week training program.	The Pro12Ala polymorphism modifies the association of physical activity and body mass changes in Polish women.
Péruse et al., 2010 [24]	*PPARG* Pro12Ala (rs1801282)	White; *n* = 481 (men 233, women 248 from 98 nuclear families), sedentary non-diabetic subjects from the HERITAGE study who finished a 20-week endurance training program. Age 36 ± 0.67 years	To investigate whether variants either confirmed or newly identified as diabetes susceptibility variants through GWAS modulate changes in phenotypes derived from an intravenous glucose tolerance test (IVGTT) in response to an endurance training program.	Improvements in glucose homeostasis in response to regular exercise are influenced by the *PPARG* Pro12Ala variant.
Kahara et al., 2003 [25]	*PPARG* Pro12Ala	Japanese; *n* = 123, men, age 21–69 years. Age ± SD, 45.2 ± 11.6 years	To examine the association of *PPARG* gene polymorphism in Japanese healthy men with changes in insulin resistance after intervention with an exercise program.	The *PPARG* gene polymorphism may be a reliable indicator of whether exercise will have a beneficial effect as part of the treatment of insulin resistance syndrome.
Hautala et al., 2007 [21]	*PPARD* +15C/T (rs2016520) +65A/G (rs2076167)	American; *n* = 462 white subjects (223 males, 239 females) *n* = 256 black subjects (87 males, 169 females) from the HERITAGE study. Age 17–65 years	To test the hypothesis that *PPARD* gene polymorphisms are associated with cardiorespiratory fitness and plasma lipid responses to endurance training.	DNA sequence variation in the *PPARD* locus is a potential modifier of changes in cardiorespiratory fitness and plasma high-density lipoprotein cholesterol in healthy individuals in response to regular exercise.

**Table 2:** The summary of the trainability of different allele carriers. PPAR = peroxisome proliferator-activated receptor. w = week. ↑ = increase. ↓ = decrease. lbm = lean body mass. HR = heart rate.

Gene/Polymorphism	Intervention	Genotype/Allele Difference	Parameters	Study
*PPARGC1A* Gly482Ser rs8192678	9 months 3 h/w of moderate sports endurance exercise (e.g., walking, swimming)	Gly/Gly > Ser allele carriers	↑ individual anaerobic threshold (W)	Stefan et al., 2007 [17]
	10 w 3/w 60 min cycling training at a heart rate equaling 70–90% of peak oxygen uptake (VO_2_peak)	Gly/Gly > Ser allele carriers (Ser allele-no response)	↑ slow muscle fibers’ proportion	Steinbacher et al., 2015 [18]
		Gly/Gly > Ser allele carriers	↑ mitochondria activity–Complex II	Steinbacher et al., 2015 [18]
	12 w 140 min/w 20 cm bench-stepping exercise training at lactate threshold intensity	Gly/Gly > Ser allele carriers (Ser allele-no response)	↓ low-density and total lipoprotein cholesterol	Tobina et al., 2017 [19]
	10 w 3/w 45–60 min HR equaling 80–100% of the anaerobic threshold (ANT)	Gly/Gly > Ser allele carriers (Ser/Ser-no response)	↑ VO_2_peak (mL·min^−1^·kg)	Ring-Dimitriou, et al., 2014 [20]
*PPARD* rs1053049 (complete LD with rs2076167)	9 months 3 h/w of moderate sports endurance exercise (e.g., walking, swimming)	TT > C allele carriers	↑ insulin sensitivity	Stefan et al., 2007 [17]
		TT > C allele carriers	↓ fasting insulin levels	Stefan et al., 2007 [17]
*PPARD* rs2267668	9 months 3 h/w of moderate sports endurance exercise (e.g., walking, swimming)	AA > G allele carriers	↑ insulin sensitivity	Stefan et al., 2007 [17]
		AA > G allele carriers	↑ individual anaerobic threshold (W)	Stefan et al., 2007 [17]
		AA > G allele carriers (G allele-negative response)	↑ VO_2_peak (mL·min^−1^·kg lbm)	Stefan et al., 2007 [17]
*PPARD* +15C/T (rs2016520) +65A/G (rs2076167)	20 w 3/w at 55–75% of baseline VO_2_max for 30–50 min	T allele carriers > +15CC (only in black subjects)	↑ VO_2_max ↑ maximum power output	Hautala et al., 2007 [21]
		+15CC > T allele carriers +65GG > A allele carriers (only in white subjects)	↑ plasma HDL cholesterol	Hautala et al., 2007 [21]
*PPARG* Pro12Ala rs1801282	10 w 3–4/w 40 min sessions of endurance treadmill walking and stationary cycling at 65–75% of heart rate reserve	Men: Pro/Pro < Ala/Pro	↓ fasting insulin and insulin AUC following intervention	Weiss et al., 2005 [22]
	12 w 3/w 60 min at 50–75% heart rate max. aerobic	Pro/Pro > Ala allele carriers	↓ body fat	Zarebska et al., 2014 [23]
	20 w 3/w at 55–75% of baseline VO_2_max for 30–50 min	Ala carriers > Pro/Pro (Pro allele-negative response in some parameters)	↑ glucose tolerance (glucose effectiveness, acute insulin response to glucose, and disposition index)	Péruse et al., 2010 [24]
	3 months 2–3/w 2–3/day 20–60 min at 50% of the maximal heart rate of brisk walking	Ala allele carriers > Pro/Pro	↓ fasting immunoreactive insulin (IRI) ↓ homeostasis model assessment–insulin resistance index (HOMA-R)	Kahara et al., 2003 [25]

## Supplementary Materials

Supplementary materials can be found at http://www.mdpi.com/1422-0067/19/5/1472/s1.

## Abbreviations

GWAS	Genome-wide association study
PRISMA	Preferred Reporting Items for Systematic Reviews and Meta Analyses
SPIRIT	Standard Protocol Item Recommendation for Interventional Trials
PPAR	Peroxisome proliferator-activated receptor
IRI	Fasting immunoreactive insulin
HOMA-R	Homeostasis model assessment-insulin resistance index
