# Supplementary material for: The Role of Peroxisome Proliferator-Activated Receptors and Their Transcriptional Coactivators Gene Variations in Human Trainability: A Systematic Review
Source: Int J Mol Sci. 2018 May 15;19(5):1472. doi: 10.3390/ijms19051472 (PMC5983571; doi:10.3390/ijms19051472)
Supplement: Supplementary file 1 [file ijms-19-01472-s001.zip › Supplementary_material_1.pdf]

SPIRIT
STANDARD PROTOCOL ITEMS: RECOMMENDATIONS FOR INTERVENTIONAL TRIALS

SPIRIT Checklist: Adapted items to address an interventional protocol in genomic studies

| Section/item | Item No | Description |
|---|---|---|
| **Administrative information** | | |
| Title | 1 | Descriptive title identifying the interventions. |
| Interventional trial registration | 2 | Trial identifier and registry name. Date and version identifier. If not yet registered, name of intended registry |
| Funding | 3 | Sources and types of financial, material, and other support |
| Roles and responsibilities | 4 | Names, affiliations, and roles of protocol contributors analysis, and interpretation of data; writing of the report; and the decision to submit the report for publication, including whether they will have ultimate authority over any of these activities |
| **Introduction** | | |
| Background and rationale | 5 | Description of research question and justification for undertaking the interventional trial, including summary of relevant studies (published and unpublished) examining benefits and harms for each intervention |
| | | Explanation for choice of comparators |
| Objectives | 6 | Specific objectives or hypotheses |
| Trial design | 7 | Justification or description of trial design including type of trial (eg, parallel group, crossover, factorial, single group), allocation ratio, and framework (eg, superiority, equivalence, noninferiority, exploratory). Possibly mentioned in methods. |
| **Methods: Participants, interventions, and outcomes** | | |
| Study setting | 8 | Description of study settings (eg, community clinic, academic hospital) and list of countries where data will be collected. Reference to where list of study sites can be obtained |
| Eligibility criteria | 9 | Inclusion and exclusion criteria for athletes/participants. If applicable, eligibility criteria for study centres and individuals who will perform the interventions (eg, surgeons, psychotherapists) |
| Interventions | 10 | Interventions for each group with sufficient detail to allow replication, including how and when they will be administered e.g.: |

| | | |
|---|---|---|
| | | Criteria for discontinuing or modifying allocated interventions for a given trial participant (eg, drug dose change in response to harms, participant request, or improving/worsening disease) |
| | | Strategies to improve adherence to intervention protocols, and any procedures for monitoring adherence (eg, drug tablet return, laboratory tests) |
| | | Relevant concomitant care and interventions that are permitted or prohibited during the trial |
| Outcomes | 11 | Primary, secondary, and other outcomes, including the specific measurement variable (eg, oxygen uptake), analysis metric (eg, change from baseline, final value, time to event), method of aggregation (eg, median, proportion), and time point for each outcome. Explanation of the clinical relevance of chosen efficacy and harm outcomes is strongly recommended |
| Participant timeline | 12 | Time schedule of enrolment, interventions (including any run-ins and washouts), athletes season, assessments, and visits for participants. A schematic diagram is highly recommended. |
| Sample size | 13 | Estimated number of participants needed to achieve study objectives and how it was determined, including clinical and statistical assumptions supporting any sample size calculations |
| Recruitment | 14 | Reporting strategies for achieving adequate participant enrolment to reach target sample size |
| **Methods: Assignment of interventions (for controlled trials)** | | |
| Blinding (masking) | 15 | Who will be blinded after assignment to interventions (eg, trial participants, care providers, outcome assessors, data analysts), and how |
| **Methods: Data collection, management, and analysis** | | |
| Data collection methods | 16 | Description for assessment and collection of outcome, baseline, and other trial data, including any related processes to promote data quality (eg, duplicate measurements, training of assessors) and a description of study instruments (eg, questionnaires, laboratory tests) along with their reliability and validity, if known. Reference to where data collection forms can be found, if not in the protocol |
| Data management | 17 | Description for data entry, coding, security, and storage, including any related processes to promote data quality (eg, double data entry; range checks for data values). Reference to where details of data management procedures can be found, if not in the protocol |
| Statistical methods | 18 | Statistical methods for analysing primary and secondary outcomes. Reference to where other details of the statistical analysis plan can be found, if not in the protocol. |

**Methods: Monitoring**

| | | |
|---|---|---|
| monitoring | 19 | Description of intervention stopping guidelines, including who will have access to these interim results and make the final decision to terminate the trial. |
| Harms | 20 | Description for collecting, assessing, reporting, and managing solicited and spontaneously reported adverse events and other unintended effects of trial interventions or trial conduct |

**Ethics and dissemination**

| | | |
|---|---|---|
| Research ethics approval | 21 | Stating research ethics committee/institutional review board (REC/IRB) approval |
| **Results** | 22 | Report the numbers of individuals at each stage of the study, completing follow-up, and analysed in results. Indicate the number of participants with missing data for each variable of interest. Explain how missing data were addressed. |
| | 23 | Report numbers of outcome events or summary measures. |
| **Biological specimens** | 24 | The collection, laboratory evaluation, and storage of biological specimens for genetic or molecular analysis in the current trial and for future use in ancillary studies, if applicable |
| **Discussion** | 25 | Summarise key results with reference to study objectives in discussion. |
| | 26 | Discuss the generalisability (external validity) of the study results. |
| | 27 | Discuss limitations of the study, taking into account sources of potential bias or imprecision. Discuss both direction and magnitude of any potential bias. |
| | 28 | Give a cautious overall interpretation of results considering objectives, limitations, multiplicity of analyses, results from similar studies, and other relevant evidence. |

*It is strongly recommended that this checklist be read in conjunction with the SPIRIT 2013 Explanation & Elaboration for important clarification on the items. Amendments to the protocol should be tracked and dated. The SPIRIT checklist is copyrighted by the SPIRIT Group under the Creative Commons "Attribution-NonCommercial-NoDerivs 3.0 Unported" license.

## S1 Table. Standard Protocol Items: Recommendations for Interventional Trials (SPIRIT) checklist

| **Item** | 1 | 2 | 3 | 4 | 5 | 6 | 7 | 8 | 9 | 10 | 11 | 12 | 13 | 14 | 15 | 16 | 17 | 18 | 19 | 20 | 21 | 22 | 23 | 24 | 25 | 26 | 27 | 28 |
|---|---|---|---|---|---|---|---|---|---|---|---|---|---|---|---|---|---|---|---|---|---|---|---|---|---|---|---|---|
| (Aksenov and Ilyin 2017) | Y | N | N | N | Y | Y | Y | Y | Y | N | N | N | Y | N | N | Y | Y | Y | N | N | N | Y | Y | N | Y | N | N | N |
| (Ghosh, Vivar et al. 2013) | Y | N | Y | Y | Y | N | Y | Y | Y | Y | N | Y | Y | Y | N | Y | Y | Y | N | Y | N | Y | Y | Y | Y | Y | Y | Y |
| (Pérusse, Ruchat et al. 2010) | Y | Y | Y | N | Y | Y | Y | Y | N | Y | Y | N | Y | N | N | Y | Y | Y | N | Y | Y | Y | Y | Y | Y | Y | N | Y |
| (Ring-Dimitriou, Kedenko et al. 2014) | Y | N | Y | Y | Y | Y | Y | Y | Y | Y | Y | Y | Y | Y | N | Y | Y | Y | Y | Y | Y | Y | Y | Y | Y | Y | N | Y |
| (Stefan, Thamer et al. 2007) | Y | Y | Y | Y | Y | Y | N | Y | Y | Y | Y | N | N | N | N | Y | N | Y | N | N | Y | Y | Y | Y | Y | Y | Y | Y |
| (Steinbacher, Feichtinger et al. 2015) | Y | Y | Y | Y | Y | Y | N | Y | Y | Y | Y | Y | N | Y | N | Y | N | Y | N | N | Y | Y | Y | Y | Y | Y | N | Y |
| (Tobina, Mori et al. 2017) | Y | Y | Y | Y | Y | Y | N | N | Y | Y | Y | Y | N | N | Y | Y | N | Y | N | N | Y | Y | Y | Y | Y | Y | Y | Y |
| (Weiss, Kulaputana et al. 2005) | Y | Y | Y | Y | Y | Y | N | Y | Y | Y | Y | Y | N | N | N | Y | N | Y | N | N | Y | Y | Y | Y | Y | N | Y | Y |
| (Zarebska, Jastrzebski et al. 2014) | Y | Y | Y | Y | Y | Y | N | N | Y | Y | Y | Y | N | N | N | Y | N | Y | Y | N | Y | Y | Y | Y | Y | N | Y | Y |
| (Hautala, Leon et al. 2007) | Y | Y | Y | Y | Y | Y | N | N | Y | Y | Y | Y | N | N | N | Y | N | Y | Y | N | Y | Y | Y | Y | Y | N | Y | Y |
| (Kahara, Takamura et al. 2003) | Y | Y | Y | Y | Y | Y | N | N | Y | Y | Y | Y | N | N | N | Y | N | Y | Y | N | Y | Y | Y | Y | Y | N | Y | N |
| (He, Hu et al. 2008) | Y | Y | Y | Y | Y | Y | N | N | Y | Y | Y | Y | N | N | N | Y | N | Y | Y | N | Y | Y | Y | Y | Y | N | Y | Y |
