# Supplementary material for: The Role of Peroxisome Proliferator-Activated Receptors and Their Transcriptional Coactivators Gene Variations in Human Trainability: A Systematic Review
Source: Int J Mol Sci. 2018 May 15;19(5):1472. doi: 10.3390/ijms19051472 (PMC5983571; doi:10.3390/ijms19051472)
Supplement: Supplementary file 1 [file ijms-19-01472-s001.zip › SupplementaryMaterial_2.pdf]

Data extraction form

Reviewer...Date..

Author..Journal....................................

Study method

Cross sectional Intervention GWAS RCT

Other:

Study aim:____

____

Participants

n =..................settings (age, height, weight,other):

population:

training program description:

Genes data

SNPs:...

Intervention details

The load description ....

Duiration...

Fenotype values:...

Authors conclusions:

____

____

____

____

Reviewer decision:

____

____

____

Created by XMLmind XSL-FO Converter.
